# Effects of Nitrogen Addition and Reproductive Effort on Nutrient Resorption of a Sand-Fixing Shrub

**DOI:** 10.3389/fpls.2020.588865

**Published:** 2020-12-15

**Authors:** Lilong Wang, Yulin Li, Yulong Duan, Jie Lian, Yongqing Luo, Xuyang Wang, Yayong Luo

**Affiliations:** Naiman Desertification Research Station, Northwest Institute of Eco-Environment and Resources, Chinese Academy of Sciences, Lanzhou, China

**Keywords:** nutrient resorption, nitrogen addition, reproductive effort, desertification, foliar nutrient concentration

## Abstract

*Caragana microphylla* is a sand-fixing leguminous shrub with strong resistance to drought, cold, and low soil fertility. As a result, it plays an essential role in combating desertification in northern China, but little is known about its nutrient budget. Nutrient resorption is a key process in plant nutrient conservation and has marked ecological implications for plant fitness and ecosystem nutrient cycling. We studied the effects of both nitrogen (N) addition and reproductive effort on leaf N resorption of *C. microphylla* in a temperate semi-arid sandy land in China. The results showed that sprouting of the early leaves from over-wintered buds employs a strategy for slow returns on nutrient investment with smaller specific leaf area (*SLA*) and higher N resorption efficiency, whereas the late leaves, which sprout from current-year buds, employ a strategy for quick returns on nutrient investment with higher SLA and lower N resorption efficiency. N addition significantly increased the N resorption efficiency from early leaves while exerting no impact on late leaves, suggesting that the increased N recovery from early leaves is done to sustain the high N demands of late leaves. Reproductive effort did not affect the N resorption from early or late leaves due to the temporal separation between fruit production and leaf senescence. Taken together, our results demonstrate that *C. microphylla* has evolved different investment strategies for leaf N in early and late leaves to conserve nutrients and facilitate its growth in desertified environments.

## Introduction

Nutrient resorption (retranslocation) in plants is the remobilization of nutrients from senescing tissues to living organs or storage structures (Aerts and Chapin, 2000). This process appears to be a key nutrient conservation mechanism in most plant species and in most ecosystems, as this process makes plants less dependent on nutrient availability from their environment (Wright and Westoby, 2003). Based on a global estimate, an average of approximately 50% of leaf nitrogen (N) is recycled via resorption (Vergutz et al., 2012). It is reasonable to expect that plants that inhabit infertile environments would be more efficient at resorbing nutrients than plants that inhabit fertile environments (Li et al., 2013). However, this hypothesis is still being debated, as it has been supported by some studies (Killingbeck, 1993b; Wright and Westoby, 2003; Yuan et al., 2005) but contradicted by others (Killingbeck and Whitford, 2001; Wang et al., 2017). Such debate suggests that nutrient resorption is not a simple function of soil fertility, especially for plant species that are adapted to nutrient-poor environments (Drenovsky et al., 2010; Huang et al., 2018).

Desertification is both a natural process and a human-induced form of land degradation process. The anthropogenic form occurs primarily in the fragile ecosystems of arid and semi-arid climatic regions, and leads to substantial loss of topsoil nutrients and, subsequently, decreased plant species diversity and vegetation cover (Su and Zhao, 2003). In China, some shrub species that have adapted to low-nutrient soils have been selected and widely planted in areas with mobile sands to control desertification, and leguminous species have been more successful than non-leguminous species due to their N-fixing ability (Dong et al., 2009; Ala et al., 2014). Leguminous species generally resorb less N from their leaves than non-leguminous species, as the N-fixing symbionts provide the leguminous species with available N through biochemical fixation and make them less dependent on soil N availability (Stewart et al., 2008; Vergutz et al., 2012). A literature review including global woody plant data set revealed that N fertilization decreases leaf N resorption efficiency by an average of 12% (Yuan and Chen, 2015). However, the responses of leaf nutrient resorption by leguminous species in sandy land to N addition are still unclear.

In addition to being a key element that limits vegetative growth, N is also an essential element in reproduction. As a result, N availability has important impacts for reproductive effort (Barker and Sawyer, 2005), which is defined as the allocation of resources such as energy and nutrients to reproductive structures rather than to vegetative structures (Thompson and Stewart, 1981). In addition to soil nutrient availability, the reproductive effort is a primary driver of plant nutrient uptake and use, yet few studies have addressed the influence of reproductive tissues on nutrient resorption (Tully et al., 2013; Peng et al., 2019), and our literature review found no study of this factor for sand-fixing leguminous species.

*Caragana microphylla* is a deciduous leguminous shrub that is widely distributed in the arid and semi-arid sandy lands of northern China (Zhang et al., 2006). As a pioneer shrub species for vegetation re-establishment, *C. microphylla* is well adapted to nutrient-poor sandy soils and resistant to drought, wind erosion, and sand burial; these characteristics let it play vital roles in fixation of mobile sands and in soil rehabilitation (Cao et al., 2004). Much of the research on *C. microphylla* plantations focused on physiological traits (Zhang et al., 2006), water use (Yue et al., 2008), soil properties (Su and Zhao, 2003), and soil microbial activity (Liu et al., 2013). We found only one study that examined the nutrient conservation traits (Li et al., 2013). *Caragana microphylla* has two leaf emergence peaks: in late April, the early leaves emerge shortly after bud burst from winter buds that formed during the previous growing season and reach full maturity in late June. In contrast, late leaves emerge from buds produced during the current growing season, after maturation of the early leaves. They mature in August. In mid-September, most of the early leaves turn yellow and senesce, whereas the late leaves remain green. The late leaves are generally shed abruptly in response to cold weather in mid- to late-October. To the best of our knowledge, the difference in nutrient resorption characteristics for early and late leaves of *C. microphylla* has not been reported. In addition, it is currently unknown how N addition and reproductive effort affect nutrient resorption by the early and late leaves.

We designed the present study to investigate how N supply and reproductive effort affect leaf nutrient status and nutrient resorption by *C. microphylla*, a deciduous leguminous shrub in the semi-arid sandy land of northern China. We tested the separate and combined effects of N addition and decreased sink treatments on *C. microphylla*. We hypothesized that (1) the chemical and morphological traits of early and late leaves would differ, resulting in different leaf N budget characteristics; (2) the N resorption efficiency from early leaves would be higher than that from late leaves; (3) N addition would lead to decreased N resorption from both early and late leaves; and (4) reproductive effort would affect the N resorption from early and late leaves.

## Materials and Methods

### Study Area and Experimental Setup

This study was conducted at the Naiman Desertification Research Station (42°58′N, 120°43′E; 360 m a.s.l.), which is located in northeastern Inner Mongolia, in northern China. This region has a temperate continental semi-arid monsoonal climate, with a warm summer and a markedly cold winter. The long-term mean annual temperature is approximately 7.0°C, with monthly mean temperatures ranging from −13.1°C in January to 23.7°C in July. The long-term mean annual precipitation is 343 mm, about 80% of which occurs in the growing season from May to September (Luo et al., 2020). The wind is an important erosive force in this region, with a mean annual wind speed that ranges from 3.4 to 4.1 m s^–1^. The landscape of this region is characterized by dunes alternating with gently undulating lowland areas (Liu et al., 2013). The field experiment was carried out in a natural area of fixed sand dunes, which has been fenced since 2001 to prevent grazing by large animals. The soils are classified as Cambic Arenosols of sandy origin in the FAO soil taxonomy (Luo et al., 2020). Soil organic carbon, total N, and total P concentrations for the top 20 cm are 8.2, 0.7, and 0.3 g kg^–1^, respectively. Soil available N and available P concentrations for the top 20 cm are 10.1 and 3.8 mg kg^–1^, respectively (Li et al., 2013). *Caragana microphylla* is the dominant leguminous shrub, and the herbaceous vegetation is mainly composed of *Setaria viridis*, *Cleistogenes squarrosa*, *Corispermum mongolicum*, and *Echinops gmelinii*.

To determine the effects of N addition, reproductive effort, and their interactions on foliar nutrient resorption, we combined three levels of N addition (0, 56, and 112 g N individual^–1^, respectively), with two levels of sink decrease [flowers and fruits produced naturally or all flowers removed (FR)]. There were six treatment combinations: a control with no N addition or flower removal (N0); addition of 56 g N per individual (N1); addition of 112 g N per individual (N2); with no N addition but with sink decrease (N0 + FR); addition of 56 g N per individual with sink decrease (N1 + FR); and addition of 112 g N per individual with sink decrease (N2 + FR). All treatments were performed on randomly selected adult individuals of *C. microphylla*. We created 11 replicates for the N0, N1, and N2 treatments and 6 replicates for the N0 + FR, N1 + FR, and N2 + FR treatments, respectively. Overall, we selected 51 similar-sized adult *C. microphylla* shrubs in the study area. The selected adult individuals had no neighboring plants present within a 4 m^2^ area centered on the shrub, thereby preventing the soil resources from overlapping with other treatments.

**FIGURE 1:**
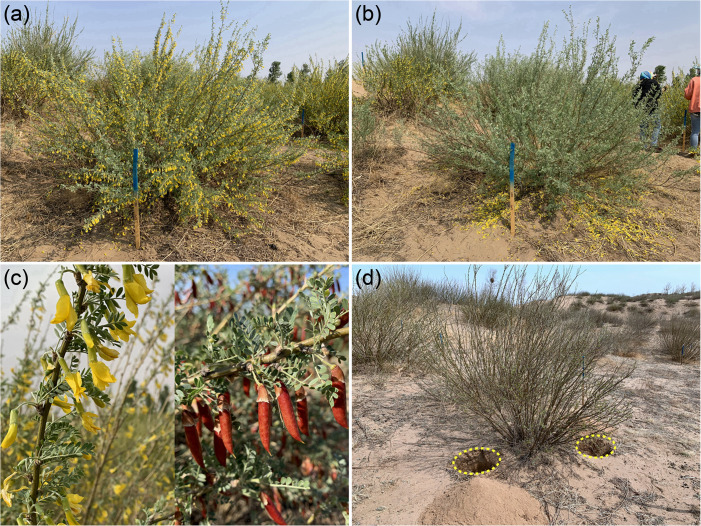
Experimental plots used in the sink decrease and nitrogen addition treatments at the Horqin Sandy Land experimental site. **(a,b)** Sink decrease was achieved by removing all flowers from each adult individual of *C. microphylla* in this treatment during the initial flowering period (Figure 2). **(c)** In contrast, the other individuals of *C. microphylla* produced fruit naturally during the growing season. **(d)** N addition was achieved by excavating two 40 cm-deep holes on opposite sides of the canopy of each individual, mixing of urea into the excavated soil, and replacing the soil in the holes. The photos were taken by the authors in 2019.

**FIGURE 2:**
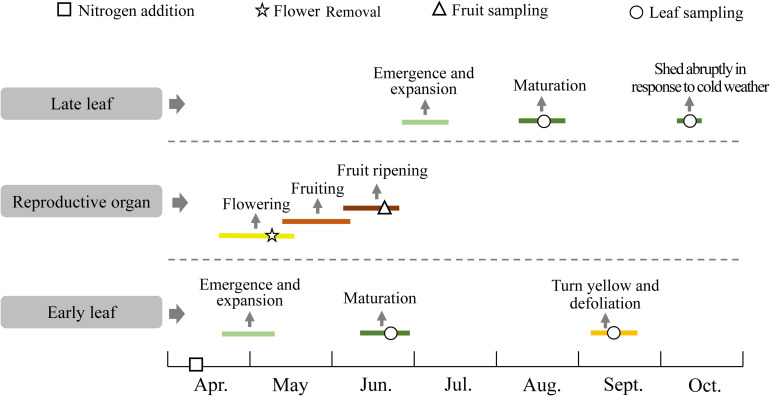
The phenology of *Caragana microphylla* and the experimental treatment and sampling dates.

Nitrogen was added as urea in spring in two 40 cm-deep holes on opposite sides of the canopy of each individual (Figure 1). The urea was mixed with the excavated soil and the soil was then replaced in the holes. Sink decrease was achieved by removing all flowers from each individual during the initial flowering period in early May, and thereafter, we monitored the plants every 2 days to remove any new flowers. The individuals that were not included in the sink decrease treatment produced flowers and fruits naturally during the growing season (Figures 1, 2).

### Field Sampling and Measurements

Mature early leaves were collected in late June 2019, and mature late leaves were collected in mid-August 2019 (Figure 2). Fully sunlit mature green leaves were collected randomly from each replicated individual (Supplementary Figure S1). In mid-September, the early leaves began to senescence, but the late leaves remained green. From this stage on, leaves of the treated individuals were checked every 3 days. To avoid underestimation of the N concentration in senesced early leaves caused by decomposition, we collected fully senesced leaves that were still attached to the stem but that had formed an abscission layer at the base of the petiole. These leaves were easily identified by their yellow color, and by the fact that they were easily removed from the plant by gently flicking the branch or leaf with a finger (Supplementary Figure S2). Generally, cold weather begins in mid-to late October and causes immediate abscission of the late leaves, which are not fully senesced. In mid-October 2019, we collected the late leaves within 1 day after leaf fall (Figure 2). We collected 10 g of fresh leaf samples from each individual, and stored half of the samples in an icebox to keep them fresh for morphological analysis, and stored the rest in paper envelopes for nitrogen analysis. The appearance of flower buds of *C. microphylla* coincides with initial expansion of the early leaves (between late April and early May). The fruit ripens in early summer (late June), with the pods turning dark brown and dry. We selected eight branches on each individual and collected all the fruits (including seeds and pods) and leaves on branches with the same length (50 cm) and measured the dry weight.

Both leaf and fruit samples (with fruits divided into seeds and pods) were oven-dried at 70°C to a constant weight and ground into a fine powder using a ball mill (MM200, Retsch, Haan, Germany) to enable chemical analysis. The N concentration was measured using an elemental analyzer (ECS4010, Costech Analytical, Valencia, CA, United States). The fresh leaf samples stored in the icebox were transported to the laboratory where the leaf area (*A*_*f*_) was determined immediately using a photographic method (Pérez-Harguindeguy et al., 2013), in which digital images were analyzed using the ImageJ software^1^. We then oven-dried the fresh leaf samples at 70°C to a constant weight (*M*_*d*_). We calculated the specific leaf area (*SLA*) as *A*_*f*_/*M*_*d*_ (cm^2^ g^–1^).

### Calculations and Statistical Analysis

Leaf N resorption efficiencies (NRE) were quantified as the proportional withdrawal of N during senescence (Lü et al., 2013) and was calculated as follows:

$$N\,R\,E=\frac{N_{\text{green}}-N_{\text{senesced}}}{N_{\text{green}}}\times 100\%$$

where *N*_*green*_ and *N*_*senesced*_ are mass-based N concentrations in green and senesced leaves, respectively. In this study, we calculated the N resorption efficiency from early and late leaves.

Data were tested for normality using the Kolmogorov-Smirnov test and for the homogeneity of error variance using Levene’s test. The impacts of N addition and sink decrease and their interaction on leaf N concentration and resorption efficiency were analyzed by means of two-way ANOVA. We used one-way ANOVA to test for differences of early and late leaf traits and fruit traits at different N addition level. We used independent-sample *t*-tests to test for differences of leaf traits between the early and late leaves, as well as differences of fruit traits between the seeds and pods. We used Pearson’s correlation coefficient (*r*) to identify significant relationships between leaf and fruit traits. All analyses were performed in SPSS 20.0 (SPSS Inc., Chicago, IL, United States).

## Results

### Differences of Leaf Traits Between Early and Late Leaves

**FIGURE 3:**
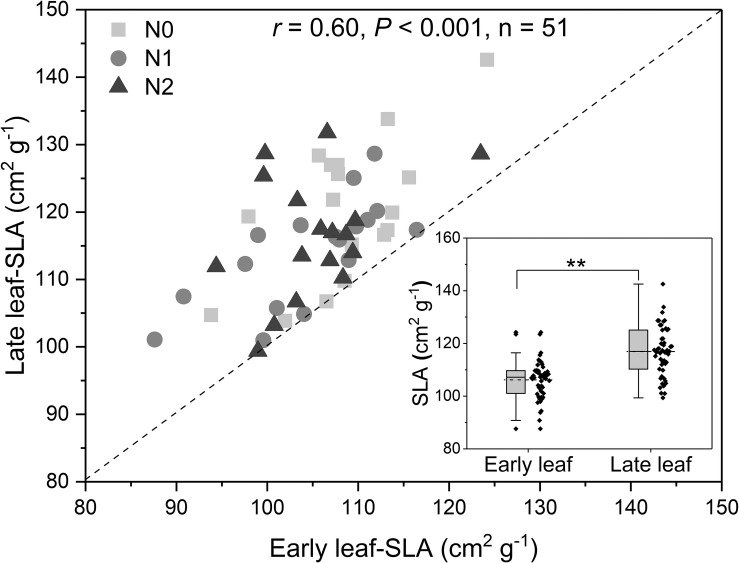
Relationship between specific leaf area (*SLA*) of early leaves and late leaves, and (inset) differences in *SLA* between the leaf types (In the box plots, the horizontal line represents the median, the box represents the 25–75% interval, whiskers represent the 95% confidence interval, and dots outside this range represent outliers; ** represent significant at *P* < 0.01, *t*-test). The *r*-value is Pearson’s correlation coefficient. N addition (*n* = 17 for each treatment): N0 (control), with no N addition; N1, 56 g N per individual; N2, 112 g N per individual. The dashed line is *y* = *x*. The points represent *SLA*-values for the same individual plants; that is, each point represents the mean *SLA* for all sampled leaves from that plant.

**FIGURE 4:**
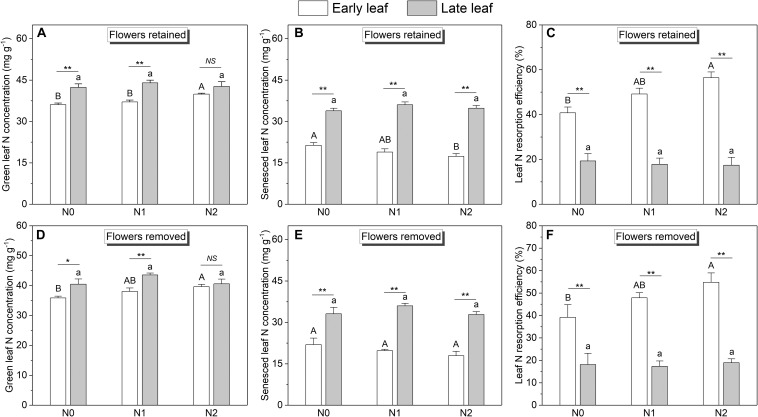
N concentrations and resorption efficiencies of the early leaves and late leaves at different N addition levels (*n* = 11 for plants with flowers, *n* = 6 for plants with flower removal): N0 (control), with no N addition; N1, 56 g N per individual; N2, 112 g N per individual. Values are means ± SE. **(A–C)** Represent plants without sink decrease treatment (Flowers retained). **(D–F)** Represent plants with sink decrease treatment (Flowers removed). Bars labeled with different letters differ significantly (one-way ANOVA) between N addition treatments (*P* < 0.05) for early leaves (upper-case letters, i.e., A, B) and late leaves (lower-case letters, i.e., a, b), respectively. * and ** indicate significant difference at *P* < 0.05 and *P* < 0.01 level respectively (*t*-test) between the two leaves in a given treatment.

Across all treatments, the *SLA* of late leaves was significantly higher than that of early leaves (Figure 3 and Supplementary Figure S3). The N concentration in green late leaves was significantly higher than that in early leaves in the N0 and N1 treatments, but there was no significant difference in the N2 treatment (Figures 4A,D). Similarly, the N concentration of senesced late leaves was also significantly higher than that of early leaves, but the difference was significant in all treatments (Figures 4B,E). In contrast, the N resorption efficiency from late leaves was significantly lower than that from early leaves across all N treatments (Figures 4C,F).

### Responses of Leaf and Fruit Traits to N Addition and Reproductive Effort

The effects of N addition on leaf N concentration and N resorption efficiency differed between early and late leaves. For early leaves, N addition significantly increased the green leaf N concentration (Figures 4A,D), but significantly decreased the senesced leaf N concentration (Figures 4B,E). Consequently, the N resorption efficiency from early leaves was significantly enhanced by N addition (Figures 4C,F). In contrast, for late leaves, N addition did not significantly affect the leaf N concentration or N resorption efficiency.

**FIGURE 5:**
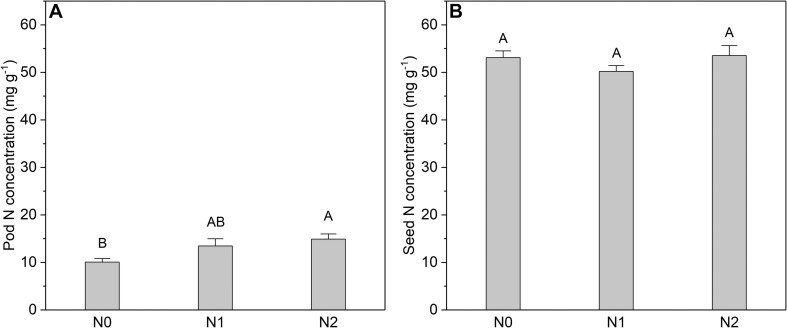
N concentrations in **(A)** pods and **(B)** seeds at different N addition levels. Values are means ± SE. N addition (*n* = 6 for each treatment): N0 (control), with no N addition; N1, 56 g N per individual; N2, 112 g N per individual. In each graph, bars labeled with different letters i.e., A, B differed significantly between treatments.

**FIGURE 6:**
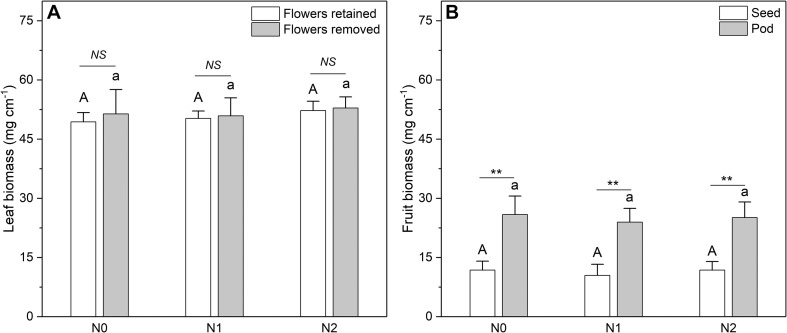
Biomass of **(A)** leaves and **(B)** fruits at different N addition levels. N addition [*n* = 6 for plants with sink decrease (Flowers removed); *n* = 11 for plants without sink decrease (Flowers retained)]: N0 (control), with no N addition; N1, 56 g N per individual; N2, 112 g N per individual. Values are means ± SE. Bars labeled with different letters differ significantly between N addition levels for **(A)** plants without flower removal (upper-case letters, i.e., A, B) and with flower removal (lower-case letters i.e., a, b) for leaf biomass and **(B)** between seed biomass (upper-case letters, i.e., A, B) and fruit biomass (lower-case letters i.e., a, b). In **(A)**, differences between sink decrease treatments were not significant; in **(B)** seeds and pods differed significantly (**one-way ANOVA, *P* < 0.01). For differences at a given N addition level, NS represents non-significance; **represents a significant difference (*t*-test, *P* < 0.01).

The N concentration of the fruits was also affected by N addition (Figure 5). However, only the pod N concentration increased significantly with increasing N input, whereas the seed N concentration showed no response. In contrast to N concentration, the biomass of leaves and fruits was not significantly affected by N addition (Figure 6). The sink decrease treatment had no significant effect on the N concentration or N resorption efficiency from early and late leaves (Table 1), and we found no significant N addition × flower removal (sink decrease) interaction for the N concentration of green and senesced leaves or for the N resorption efficiency between early and late leaves (Table 1). Moreover, neither N addition nor sink decrease significantly affected leaf biomass (Figure 6A).

**TABLE 1:** The significance results (*P*-values) for two-way ANOVA for the effects of N addition (N) and sink decrease (FR, flower removal) on leaf N concentration and N resorption efficiency (*NRE*).

Source	N in green leaves		N in senesced leaves		*NRE*	
	Early leaf	Late leaf	Early leaf	Late leaf	Early leaf	Late leaf
N	**< 0.01**	0.23	**0.01**	0.10	**< 0.01**	0.94
FR	0.78	0.23	0.54	0.36	0.56	0.99
N × FR	0.60	0.83	0.99	0.74	0.99	0.93

*N, nitrogen addition (n = 17); FR, sink decrease by flower removal (n = 6); NRE, N resorption efficiency.EL and LL represent early and late leaves, respectively. Significant effects (*P* < 0.01 level) are boldfaced.*

### Correlations Between Traits

Across all treatments, the N concentrations of seeds and pods were not significantly correlated with the N resorption efficiency from early and late leaves (all *P* > 0.05, Supplementary Figure S4). We found the N resorption efficiency from early and late leaves were significantly positively correlated with the N concentration of the corresponding green leaves (Table 2). In contrast, the N resorption efficiency from early and late leaves was significantly negatively correlated with the N concentration of the corresponding senesced leaves (Table 2). The *SLA*-values for early and late leaves on the same plant were significantly positively correlated (Figure 3 and Table 2). However, there was no correlation between *SLA* and the leaf N concentration or the N resorption efficiency for both early and late leaves (Table 2).

**TABLE 2:** Pearson’s correlation coefficient (*r*) matrix for the different leaf traits of early and late leaves.

	Early leaves				Late leaves			
	*N*_*green*_	*N*_*sen*_	*NRE*	*SLA*	*N*_*green*_	*N*_*sen*_	*NRE*	*SLA*
Early leaves								
*N*_*green*_	1	−0.06	0.36**	−0.06	−0.05	0.16	−0.20	0.03
*N*_*sen*_		1	−0.95**	0.21	0.00	0.22	−0.18	0.16
*NRE*			1	−0.22	−0.01	−0.15	0.11	−0.14
*SLA*				1	0.12	−0.20	0.27	0.60**
Late leaves								
*N*_*green*_					1	0.30*	0.57**	0.04
*N*_*sen*_						1	−0.61**	−0.06
*NRE*							1	0.08
*SLA*								1

*Sample sizes for early and late leaves were n = 51. N_*green*_ and N_*sen*_ represent the N concentrations in green and senesced leaves, respectively; NRE represents the nitrogen resorption efficiency; SLA, represents the specific leaf area. Significant correlations are boldfaced; ^∗^ and ^∗∗^ represent significance at P < 0.05 and P < 0.01 level respectively.*

## Discussion

### Efficiency of N Resorption in Early and Late Leaves

Based on the emergence date, the leaves of deciduous woody species can be divided into two categories: early leaves that formed in the over-wintering buds, and late leaves that formed in the current-year buds (Wan and Sosebee, 1990). Studies have shown that in some species, the early and late leaves differ significantly in their morphological and physiological traits (Wan and Sosebee, 1990; Herrick and Thomas, 2003). However, to the best of our knowledge, the nutrient resorption characteristics of early and late leaves have mostly been overlooked. Our experiment showed that the N resorption efficiency from early leaves of *C. microphylla* was between 40 and 60%, vs. less than 20% for late leaves. On average, terrestrial plants resorb 50% of their foliar N before leaf abscission (Vergutz et al., 2012). Based on this threshold, *C. microphylla* showed efficient and inefficient N resorption in early and late leaves, respectively, and this supports our first and second hypotheses that the early and late leaves would differ in their N resorption pattern. The inefficient resorption of N in late leaves is consistent with some previous research (Killingbeck, 1993a; Tateno, 2003; Stewart et al., 2008), and could be explained by two mechanisms. First, N-fixing species often retain green leaves longer in the autumn than other species, which results in leaf abscission without full senescence when cold weather arrives (Killingbeck, 1993a; Stewart et al., 2008). This inefficient N resorption pattern may represent a trade-off between N conservation and energy assimilation by N-fixing species in temperate zones, as the amount of energy assimilated during the extended growth period is more than what is required to compensate for the N loss (Tateno, 2003). Second, N-fixing species are less dependent on foliar N resorption than non-N-fixing species because they can utilize N produced through fixation by their symbiotes (Guo et al., 2017).

However, it’s unclear why N resorption from the early leaves of *C. microphylla* is more efficient than resorption from the late leaves. It appears that the phenological period is the major cause of this difference because the early leaves of *C. microphylla* in the study area had turned yellow and had fully senesced long before the cold weather arrived. Consequently, the early leaves had enough time for their N to be resorbed during the senescence process, and did not need to make a trade-off between N conservation and energy assimilation like the late leaves. A study in a subtropical rain forest found that a deciduous tree species (*Lyonia ovalifolia*) can promote N resorption through an extended growth period (Zhang et al., 2013). Moreover, the efficient resorption of N by early leaves suggests that although *C. microphylla* can utilize N through N fixation by its symbiotes, leaf N resorption is also an essential mechanism for its N conservation, and this is important because the sandy soil in our study area is extremely deficient in plant-available N (Li et al., 2013). Overall, our results suggest that in the temperate zone, the phenological period is a major determinant of leaf N resorption characteristics in *C. microphylla*.

### N Addition Effects on Leaf N Concentration and Resorption

Our results showed that N addition increased the N concentration of green early leaves but decreased the N concentration of senesced early leaves. Thus, the N resorption efficiency from early leaves increased significantly with increasing N addition. However, N addition had no significant impact on the N resorption from late leaves. These results contradict our third hypothesis, that N addition would have negligible effects on leaf N resorption. In general, N addition can disproportionately increase N concentrations in both green and senesced leaves, and consequently, decrease N resorption efficiency at the end of the growing season (Kozovits et al., 2007; Li et al., 2010; Lü et al., 2013; Yuan and Chen, 2015; Du et al., 2017; Zhang et al., 2019), suggesting that soil nutrient availability is an important factor in leaf nutrient resorption. However, for *C. microphylla*, the N demand of late leaves rather than soil nutrient availability may be the main factor that affects N resorption from early leaves. To construct new leaves, a plant must obtain N from the soil or via resorption from other plant tissues, including senescent leaves (Wright and Westoby, 2003). In this study, the senescence and shedding of early leaves occurred in late summer, a time when the late leaves remain green and are functioning. In this case, the recycled N from early leaves may become less expensive to acquire than N from the soil (Mantovani et al., 2017). Since the later-produced late leaves have a significantly higher N concentration and larger leaf area than the early leaves, the N translocation may promote the growth of *C. microphylla* by helping the plant to maintain a high assimilation rate, as an increase in the area-based N concentration increases the photosynthetic capacity if other factors are not limiting (Wright et al., 2004; Mantovani et al., 2017). Therefore, it is reasonable to expect that there is N translocation between early and late leaves, but additional research (e.g., an isotope tracer experiment) will be required to support this hypothesis.

Interestingly, we found that N addition increased the N concentration of both leaves (Figure 4) and fruits (Figure 5) but did not affect their biomass (Figure 6). This could be explained by luxury consumption, as N fertilizer often results in excessive accumulation of N in vegetative and reproductive organs (i.e., an increase greater than the plant can benefit from) but a small contribution to biomass increase (Chapin, 1980). This suggests that a soil N deficiency may not be the key limiting environmental factor that restricts the growth of *C. microphylla* in our study area. This hypothesis is supported by the fact that *C. microphylla* can acquire sufficient N through fixation by its symbiotes, as the average N concentration of its mature leaves was 39 mg g^–1^ (plants with no fertilizer), which is close to the upper limit of the N concentration (15–40 mg g^–1^) that is required to maintain healthy plant growth (White and Brown, 2010). Since we only have 1 year of data, further study will be needed to determine the response of leaf and fruit biomass of *C. microphylla* to long-term N addition.

### Reproductive Effort Does Not Affect N Resorption From Early and Late Leaves

Variability in nutrient resorption is affected by source constraints (here, soil nutrients), but can also be driven by source–sink interactions (Chapin, and Moilanen, 1991; Pugnaire and Chapin, 1992; Tully et al., 2013). In annual plants, reproductive tissues such as flowers and fruits are the largest nutrient sinks during the reproductive growth stage, and play a leading role in determining leaf nutrient resorption (Peng et al., 2019). However, in perennial plants, phenology is a critical component of this relationship (Killingbeck, 2003). For example, fruits produced during leaf senescence should promote nutrient resorption because leaves and fruits act as nutrient sources and sinks, respectively, during this time (Tully et al., 2013). In contrast, if the fruits are produced before the leaves begin to senesce, this reproductive development may not affect nutrient resorption from the leaves because the sink strength of the fruits is temporally disconnected from the process of leaf senescence (Killingbeck, 2003). In the present study, we found no significant difference in N resorption efficiency of both early and late leaves between fruiting (plants without flower removal) and non-fruiting (plants with flower removal) plants (Figure 4 and Table 1). This contradicts our fourth hypothesis. Moreover, we found no significant correlations between the fruit N concentration and the N resorption efficiency of both early and late leaves (Supplementary Figure S4). This suggests that decreasing the sink (flowers) did not affect leaf N resorption by *C. microphylla*. This result could be due to the temporal separation between reproductive effort and leaf senescence, because the fruits of *C. microphylla* ripen in early summer (late June), when the early leaves have not yet begun to senesce and the late leaves are just ready to emerge. This suggests that while the early leaves are still photosynthetically efficient (i.e., have not begun to senesce), their role in supplying photosynthate to fruits may be more important than their role in supplying N (Wibbe and Blanke, 1995). Thus, reproductive effort added nothing to the sink strength during the process of leaf senescence and appears to have no effect on leaf N resorption by *C. microphylla*.

### Implications for the Adaptation of *C. microphylla* to Desertified Environments

Our results indicated that although the sandy soils in the study area are impoverished in N, the N concentration of both early and late leaves of *C. microphylla* was higher than the average values for most terrestrial plants. The higher leaf N concentration may be attributed to N-fixation by the symbiotes of this leguminous species, which is an important mechanism that allows *C. microphylla* to become established on the mobile sands of our study area (Zhang et al., 2006). This is important because N is the most common limiting nutrient worldwide, but is especially limited in desert environments (Vitousek and Howarth, 1991). In addition, the early and late leaves of *C. microphylla* differ significantly in their chemical and morphological traits, which may help it adapt to the desertified environment. From the perspective of the spectrum of plant leaf economics, the early leaves have a lower N concentration and lower *SLA* than late leaves, and therefore employ a strategy for slow returns on nutrient investment (Wright et al., 2004). This strategy helps *C. microphylla* to cope with the environmental stresses in the early spring, such as cold, drought, and strong wind erosion. In contrast, the late leaves tends to employ a strategy for quick returns on nutrient investment (Wright et al., 2004), with a higher N concentration and *SLA* than the early leaves. Because the late leaves are produced in early summer, when the moisture and temperature conditions are better for growth, the “quick-return” strategy helps *C. microphylla* to maintain efficient photosynthesis. The later phenology of the late leaves can also help the plant maintain net carbon gain and energy assimilation from photosynthesis during the early autumn (Tateno, 2003).

Nutrient resorption controls stand-level biogeochemical cycling via its effect on nutrient concentrations in senesced leaves, which in turn, have a feedback effect on litter decomposition rates and soil nutrient availability (Tully et al., 2013). In the present study, the inefficient N resorption resulted in abnormally high N concentration in senesced late leaves, which in turn may promote the decomposition of litter composed of late leaves and increase the soil N availability to the plants and soil microbes. Therefore, plant–soil feedback mediated by nutrient resorption leads to the “fertile island” effect within stands of *C. microphylla* (Yang et al., 2011; Kondo et al., 2012), which promotes the growth of herbaceous plants and improves the species diversity and ecosystem productivity in the desertified environment.

## Conclusion

In this study, we showed that the two types of leaves produced by *C. microphylla* differ significantly in their chemical and morphological traits. The early leaves have a lower N concentration and lower *SLA*, and tend to employ a “slow-return” strategy, whereas the late leaves have a higher N concentration and higher *SLA*, and tend to employ a “fast-return” strategy. Compared with the average value for terrestrial plants, the early leaves showed efficient N resorption, whereas N resorption by the late leaves was inefficient. The inefficient N resorption in late leaves results in higher litter N concentrations and may promote litter decomposition rates and increase the available soil N. N addition increased the N resorption efficiency from early leaves but had no significant impact on late leaves, which suggests that the N recovery from the early leaves supports the high demand for N by the late leaves. Reproductive effort did not affect N resorption in early or late leaves, probably due to the temporal separation between fruit production and leaf senescence. In summary, the contrasting leaf traits in early and late leaves enable *C. microphylla* to better adapt to the harsh climate and soil environment of the study area. These findings also have clear implications for understanding the regulation of nutrient resorption by N-fixing shrubs in cold-temperate desertified environments.

## Data Availability Statement

The raw data supporting the conclusions of this article will be made available by the authors, without undue reservation, to any qualified researcher.

## Author Contributions

LW and YL conceived and designed the experiments. LW, YD, and JL performed the experiments. LW, XW, and YL analyzed the data. LW wrote the manuscript. All authors contributed to the article and approved the submitted version.

## Conflict of Interest

The authors declare that the research was conducted in the absence of any commercial or financial relationships that could be construed as a potential conflict of interest.

## Footnotes

1 http://imagej.nih.gov/ij
