# Supplementary material for: Effects of Nitrogen Addition and Reproductive Effort on Nutrient Resorption of a Sand-Fixing Shrub
Source: Front Plant Sci. 2020 Dec 15;11:588865. doi: 10.3389/fpls.2020.588865 (PMC7769775; doi:10.3389/fpls.2020.588865)
Supplement: Supplementary file 1 [file Table_1.DOCX]

**Effects of nitrogen inputs and reproductive effort on nutrient resorption of a sand-fixing shrub**

Lilong Wang^1^, Yulin Li^1*^, Yulong Duan^1^, Jie Lian^1^, Yongqing Luo^1^, Xuyang Wang^1^, Yayong Luo^1^

^1^Naiman Desertification Research Station, Northwest Institute of Eco-Environment and Resources, Chinese Academy of Sciences, Lanzhou, China

^*^Corresponding author: Yulin Li

Address: Naiman Desertification Research Station, Northwest Institute of Eco-Environment and Resources, Chinese Academy of Sciences, Lanzhou, China

E-mail address: liyl@lzb.ac.cn


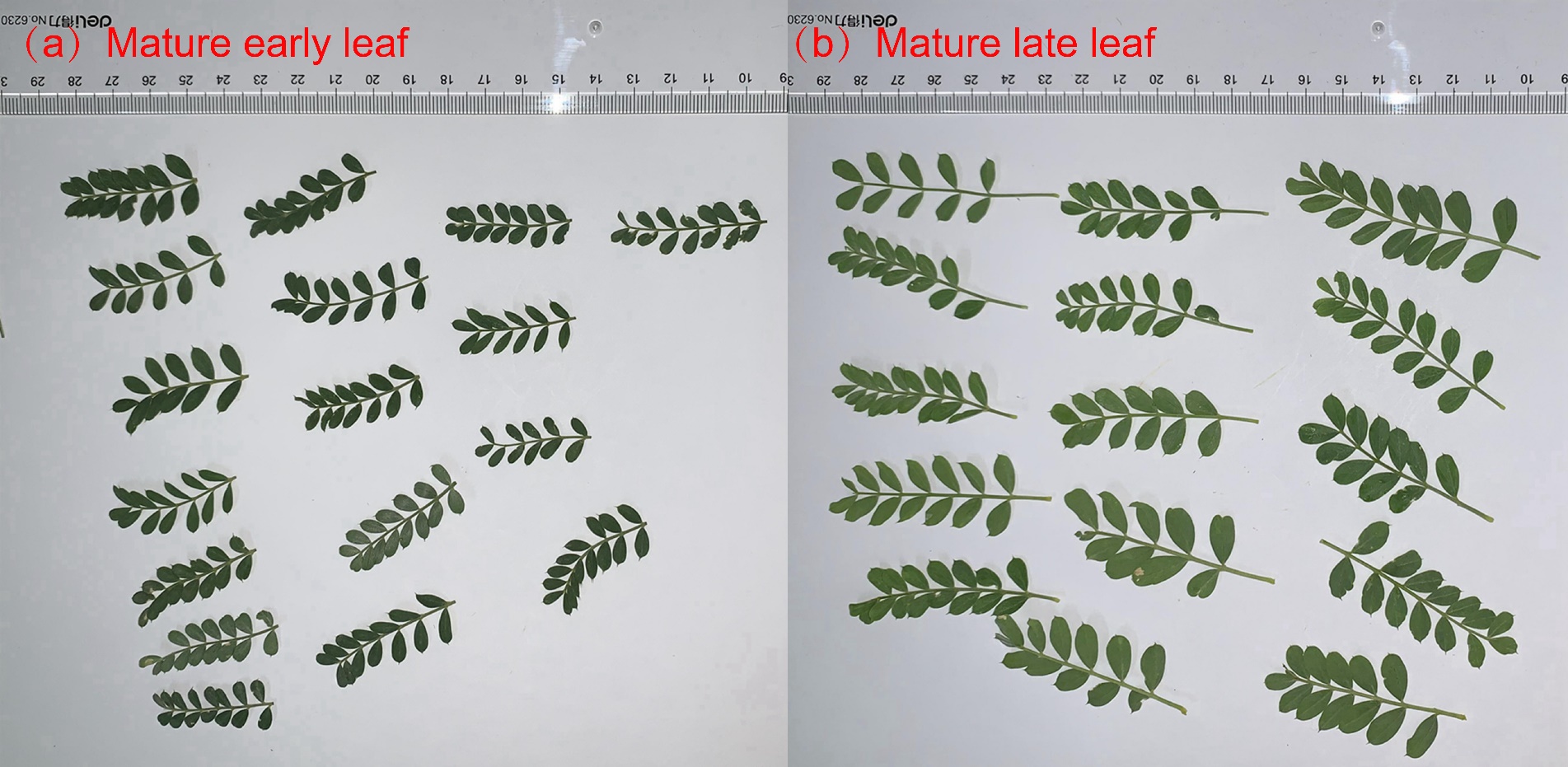


**Fig. S1** Examples of the mature (a) early and (b) late leaves of *C. microphylla*. The leaves were collected from a single individual and photographed at the same scale. The leaf area of the late leaves is clearly larger than that of the early leaves. The early leaves were photographed in 25 June 2019 and the late leaves were photographed in 18 August 2019.


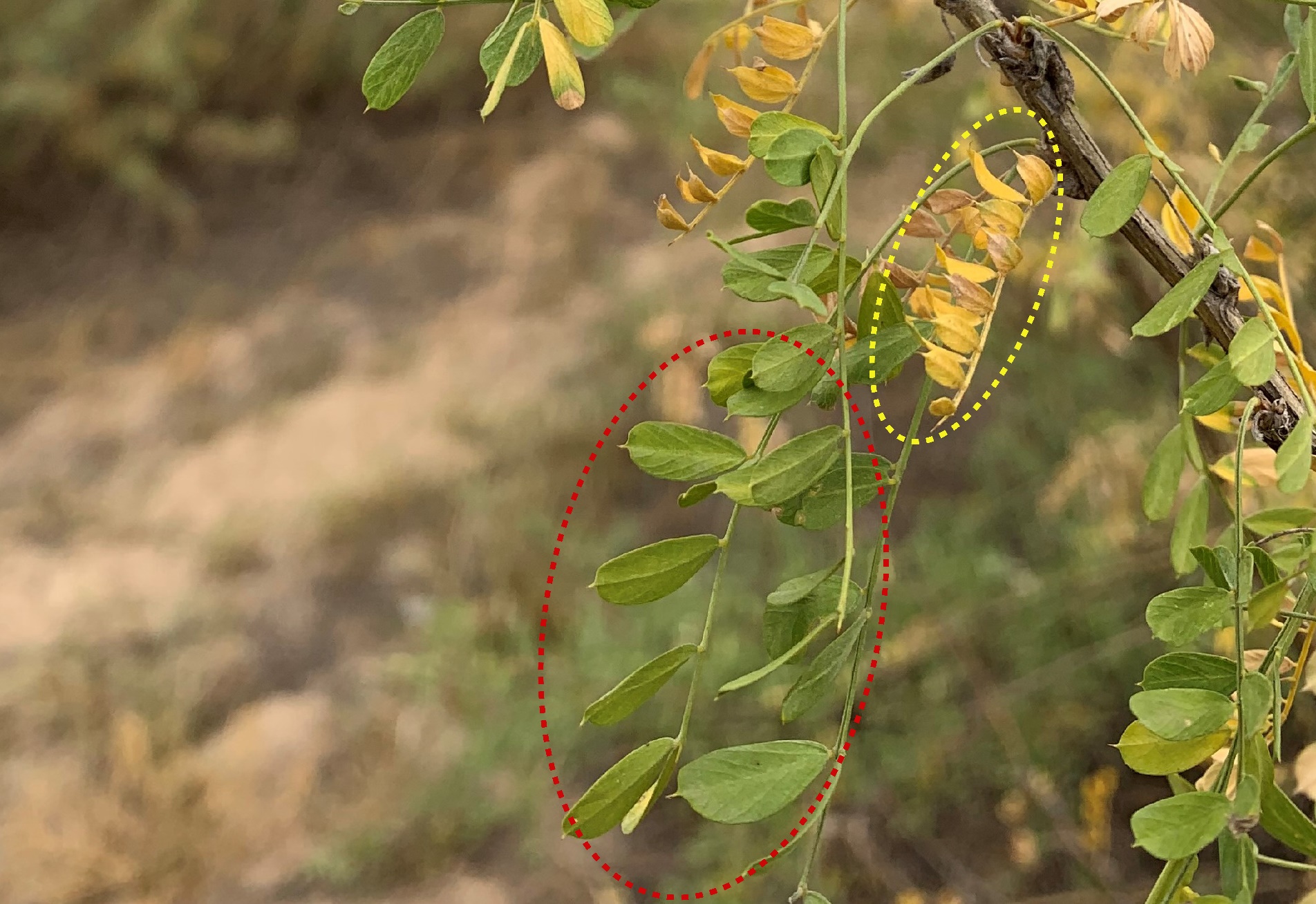


**Fig. S2** Examples of senesced early leaves. The photo was taken in mid-September 2019 in the study area. The yellow dotted circle indicates fully senesced early leaves, which are yellow and easily detached from the plant by gently flicking the branch or leaf with a finger. At this time, the late leaves were still green (the red dotted circle) and cannot be removed the same way. Most of the late leaves will stay green until mid-October and then be shed abruptly in response to cold weather.





**Fig. S3** Specific leaf area (*SLA*) of early leaves and late leaves at different N addition levels: N0 (control), with no N addition; N1, 56 g N per individual; N2, 112 g N per individual. (a) Plants without sink decrease (Flowers retained, n = 11); (b) plants with sink decrease (Flowers removed, n = 6). Bars labeled with different letters differ significantly between N addition treatments (one-way ANOVA, *P* < 0.05) for early leaves (uppercase letters) and late leaves (lowercase letters). ** indicates a significant difference (*t*-test, *P* < 0.05) between the two leaf types in a given treatment.





**Fig. S4** Relationships between leaf N resorption efficiency (*NRE*) from early leaves and late leaves and the N concentrations of seeds and pods. N addition (*n* = 11 for each treatment): N0 (control), with no N addition; N1, 56 g N per individual; N2, 112 g N per individual. The Pearson’s correlation coefficient (*r*) for each relationship is shown for each graph; the correlations were all weak and not statistically significant. The vertical dashed lines represent 50% resorption of N.
